# Nasal Mucociliary Clearance Assessment: Historical Evolution, Methodological Approaches, and the Role of Activated Charcoal as a Visible Marker

**DOI:** 10.7759/cureus.115026

**Published:** 2026-08-23

**Authors:** Stanimir D Dinev, Teodor D Atanasov

**Affiliations:** 1 Department of Otorhinolaryngology, University Hospital Sofiamed, Sofia, BGR; 2 Department of Otorhinolaryngology, University Hospital Burgasmed, Burgas, BGR; 3 General Surgery, University Hospital Sofiamed, Sofia, BGR

**Keywords:** activated charcoal test, mucociliary transport, nasal endoscopy, nasal mucociliary clearance, pediatric otorhinolaryngology, saccharin test, scintigraphy, visible marker methods

## Abstract

Nasal mucociliary clearance assessment has evolved over more than a century through a continuous search for methods that balance physiological relevance, clinical practicality, objectivity, cost, reproducibility, and patient tolerance. Early approaches were based on direct observation and dye-based techniques, followed by saccharin transit time testing, visible particle methods, charcoal-based tracers, radiolabeled particle techniques, scintigraphic assessment, and modern specialized tests of ciliary function.

The available methods differ substantially in their principles and practical applications. Saccharin transit time testing became widely used because of its simplicity, low cost, and accessibility, but its endpoint depends on subjective taste perception and patient reporting. Dye-based and visible particle techniques attempted to make mucociliary transport more directly observable, whereas radiolabeled and scintigraphic methods introduced greater objectivity at the cost of technical complexity and limited routine applicability. Modern investigations such as ciliary beat frequency analysis, high-speed video microscopy, nasal nitric oxide measurement, transmission electron microscopy, immunofluorescence, and genetic testing are essential in selected diagnostic contexts, particularly in primary ciliary dyskinesia, but they are not equivalent to direct nasal transport-time assessment.

Activated charcoal should be understood within this broader historical and methodological evolution. It is not a newly introduced method and should not be presented as a universal replacement for established techniques. Rather, it is a topical, low-cost, dark, visible marker that may permit examiner-observed recognition of the endpoint. However, the additional value of standardized endoscopic control requires prospective comparative validation. This review examines the historical development of nasal mucociliary clearance assessment, compares the major methodological approaches, and critically discusses the potential methodological role of activated charcoal as a visible marker, particularly in pediatric otorhinolaryngology.

## Introduction and background

Nasal mucociliary clearance is a fundamental functional process of the upper airway and contributes to the removal of inhaled particles, pathogens, allergens, and secretions from the nasal cavity and nasopharynx. Although this mechanism depends on coordinated ciliary activity, mucus properties, epithelial integrity, hydration, and local environmental conditions, the present review does not aim to provide an extensive physiological discussion of mucociliary clearance. Instead, its focus is historical and methodological, examining how nasal mucociliary clearance has been assessed over time, how different methods emerged, and why each technique occupies a distinct practical role [1-4].

The assessment of nasal mucociliary clearance has attracted scientific interest because it provides functional information that anatomical and airflow-based examinations cannot fully capture. Nasal endoscopy, imaging, rhinomanometry, and acoustic rhinometry may describe obstruction, structure, or airflow, but they do not directly measure the transport of material along the nasal mucosa. Consequently, methods designed to assess mucociliary transport have remained important in clinical research, respiratory physiology, rhinology, and pediatric otorhinolaryngology.

Historically, the field has evolved through repeated attempts to solve the same methodological problem: how to measure a dynamic biological transport process in a manner that is sufficiently accurate, reproducible, simple, inexpensive, and acceptable to patients. Early approaches relied on direct observation and dye-based techniques. Later, saccharin transit time testing became widely used because of its simplicity and low cost. Other methods, including visible particles, charcoal-based tracers, radiolabeled particles, rhinoscintigraphy, and modern ciliary diagnostic techniques, were introduced to improve visualization, objectivity, quantification, or diagnostic depth.

These methods are related, but they are not interchangeable. Some tests assess nasal mucociliary transport time directly by following the movement of an applied marker through the nasal cavity. Others evaluate ciliary beat frequency, ciliary beat pattern, nasal nitric oxide, ultrastructure, protein expression, or genetic causes of ciliary dysfunction. This distinction is important because a clinical marker-based transport test and a specialized diagnostic test for primary ciliary dyskinesia address different clinical and methodological questions.

Within this broader history, charcoal-based methods occupy a meaningful position among visible marker approaches. Their significance does not lie in novelty, as vegetable charcoal powder, charcoal-saccharin combinations, and charcoal dust have all been used historically for nasal mucociliary transport assessment. These studies support the feasibility of charcoal as a visible marker and its potential for examiner-observed endpoint recognition, but they do not establish superiority over other methods or demonstrate an additional benefit of endoscopic control. The contemporary methodological question is therefore whether procedural standardization and direct endoscopic visualization can improve reproducibility, endpoint recognition, and clinical feasibility; answering this question requires prospective comparative validation.

This review therefore aims to present the historical evolution of nasal mucociliary clearance assessment, examine the major methodological approaches in a balanced manner, and critically evaluate the potential methodological role of charcoal as a visible marker. Particular attention is given to pediatric otorhinolaryngology, where cooperation, anxiety, taste perception, and reliable verbal reporting may influence the performance of subjective tests.

## Review

Methodology

This article was designed as a narrative historical-methodological review rather than a systematic review or meta-analysis. PubMed/MEDLINE was searched from database inception through August 11, 2026. The search was organized around three thematic blocks: (1) nasal transport assessment, combining “nasal mucociliary clearance” or “nasal mucociliary transport” with terms such as “assessment,” “test,” “saccharin,” “charcoal,” “visible marker,” “rhinoscintigraphy,” and “technetium”; (2) pediatric and adenoid-related literature, combining the same transport terms with “child,” “children,” “adenoid hypertrophy,” and “adenoidectomy”; and (3) specialized ciliary diagnostics, combining “primary ciliary dyskinesia” with “nasal nitric oxide,” “high-speed video microscopy,” “ciliary beat frequency,” “transmission electron microscopy,” “immunofluorescence,” and “genetic testing.” No lower publication-date limit was applied because the historical evolution of nasal mucociliary clearance assessment was a prespecified focus of the review.

Reference lists of key reviews, guidelines, and landmark primary studies were also hand-searched to identify older reports not readily captured by contemporary keywords. Sources were selected when they directly informed the history, principle, validation, comparison, limitations, or pediatric applicability of nasal mucociliary transport assessment. Priority was given to peer-reviewed human studies, comparative methodological studies, pediatric studies, seminal historical reports, and major professional guidelines. Duplicate reports, animal-only studies, studies confined to lower-airway clearance without direct relevance to nasal assessment, and disease or intervention studies that used mucociliary clearance only as an outcome without informing measurement methodology were excluded from the core synthesis. The literature was synthesized narratively according to chronology and methodological category. Because the review was not designed as a systematic review, no PRISMA flow diagram, formal study-level risk-of-bias tool, or quantitative pooling was undertaken; this limitation is acknowledged below.

Historical development of nasal mucociliary clearance assessment

The historical development of nasal mucociliary clearance assessment reflects a gradual transition from direct observation to marker-based, particle-based, radiolabeled, imaging-based, and laboratory-based techniques. The central methodological challenge has remained consistent: how to measure nasal mucociliary transport in a way that is accurate, reproducible, clinically feasible, and acceptable to patients. Historical reviews describe early in vivo attempts to evaluate nasal mucociliary function in the late 19th century. Martius’ work in 1884, using stroboscopic observation, is often cited as an early attempt to directly observe nasal mucociliary activity. During the 1930s and 1940s, dye-based approaches introduced by Hilding and Tremble shifted the field toward marker-based assessment by placing dyes in the nasal cavity and recording their appearance in the oropharynx. These early techniques were technically limited, but they established the principle that mucociliary transport could be studied through the movement of an applied marker [4].

Earlier quantitative work, including the study by Andersen et al. on nasal clearance in monozygotic twins, contributed to the establishment of nasal clearance as a measurable physiological function [5]. The second half of the twentieth century then brought simpler clinical tests and more systematic comparisons between different methods. Puchelle et al. compared three approaches for measuring nasal mucociliary clearance in humans and supported the saccharin method as a simple screening test for detecting abnormally low nasal mucociliary clearance [6]. Sakakura et al. further demonstrated that nasal mucociliary clearance varies under different physiological and pathological conditions [7].

Visible and particle-based techniques were also developed during this period. A 1983 study assessed nasal mucociliary function using saccharin sky-blue powder and barium sulfate particles in 40 normal subjects and 200 patients with different nasal diseases, showing that visible marker approaches could be applied clinically across different nasal conditions [8]. Stanley et al. later described saccharin testing as a simple, inexpensive, and reproducible screening method for detecting abnormal mucociliary clearance [9]. Contemporary reviews continue to describe the saccharin transit time test as widely used and clinically applicable when performed under standardized conditions [10,11].

Charcoal-based methods emerged within the same search for practical visible tracers. Passali et al. reported the use of vegetable charcoal powder combined with 3% saccharin powder for determining nasal mucociliary transport time [12]. Shortly afterward, Passali and Bianchini Ciampoli studied mucociliary transport time in children aged three to 12 years using vegetal coal powder as a stained tracer, emphasizing that it was non-toxic, insoluble, and easily detected [13]. Comparative work later showed that nasal mucociliary transport time and speed may vary according to tracer properties by assessing vegetable charcoal powder, saccharin, seroalbumin-Tc99m, and pertechnetate-Tc99m in normal subjects [14].

In pediatric otorhinolaryngology, Hellin Meseguer et al. evaluated nasal mucociliary transport time in 30 children aged three to 13 years before and after adenoidectomy using the charcoal dust test [15]. Hellin Meseguer also reported nasal mucociliary transport findings in normal subjects using a charcoal powder method, supporting the historical use of charcoal-based assessment beyond pediatric adenoid disease [16]. Charcoal-based transport assessment was also applied in patients with inferior turbinate hypertrophy, septal deviation, and chronic sinusitis, indicating broader clinical use in rhinologic disorders [17]. More recently, Austero and Gelera compared saccharin testing and charcoal testing in Cureus; the present review does not duplicate that comparison, but places charcoal-based methods within the wider historical and methodological evolution of nasal mucociliary clearance assessment [18].

Radiolabeled and scintigraphic techniques continued the search for greater objectivity and quantification. Brondeel et al. evaluated the Tc99m particle test and the saccharin test in mucociliary examinations, while radioisotopic and rhinoscintigraphic methods later provided imaging-based approaches for measuring nasal mucociliary activity and transport rate [19-22]. More recent decades have expanded the field toward specialized ciliary diagnostics. Nasal nitric oxide, high-speed video microscopy, ciliary beat frequency analysis, transmission electron microscopy, immunofluorescence, and genetic testing have become important in the diagnostic work-up of primary ciliary dyskinesia, but these methods should be distinguished from clinical nasal transport-time tests because they often assess ciliary structure, ciliary function, nitric oxide biology, or genetic disease rather than directly measuring the time required for a marker to move through the nasal cavity [23,24].

Direct observation and dye-based methods

Direct observation and early dye-based methods represent the historical foundation of nasal mucociliary clearance assessment. Their major contribution was conceptual: they demonstrated that mucociliary activity could be approached as a measurable transport phenomenon rather than being regarded solely as an inferred physiological process. Dye-based techniques attempted to follow marker movement from the nasal cavity toward the oropharynx, thereby establishing the methodological principle later used by visible particles, charcoal-based tracers, and endoscopically controlled visible marker methods.

The strength of dye-based approaches was their conceptually direct visual assessment. However, they were limited by dye diffusion, variable spreading, inconsistent visualization, uncertain application volume, and difficulty in defining a precise endpoint. Without modern endoscopic control, accurate confirmation of marker placement and arrival at the endpoint was difficult. For this reason, dye-based methods are mainly important as historical and methodological predecessors of later visually guided nasal transport tests.

Saccharin transit time testing

The saccharin transit time test became one of the most influential methods for assessing nasal mucociliary clearance because it combined simplicity, low cost, minimal equipment requirements, and clinical feasibility. A small saccharin particle is placed on the nasal mucosa, and the patient reports the moment when a sweet taste is perceived. The elapsed time is then interpreted as nasal mucociliary transport time.

The advantages of the saccharin test are substantial. It can be performed in outpatient settings, does not require imaging or specialized laboratory infrastructure, and can be used in clinical or research cohorts when properly standardized. Its main limitation is the subjective nature of the endpoint. The method depends on taste perception, attention, cooperation, and verbal reporting. This limitation is particularly relevant in young children, anxious patients, individuals with altered taste sensation, or patients unable to follow instructions consistently. Therefore, saccharin testing remains useful, but its reliability depends on appropriate patient selection and a standardized testing technique.

Visible particle and non-charcoal marker methods

Visible particle and non-charcoal marker methods were developed to preserve the practicality of simple testing while improving the examiner’s ability to observe mucociliary transport directly. These methods include colored powders, dye-containing markers, barium sulfate particles, and other visible tracers. Their common principle is to visually track an applied marker rather than rely exclusively on patient-reported taste perception.

The practical value of visible marker methods lies in their potential to improve endpoint observability. However, visibility alone does not ensure reproducibility. Dyes may dissolve, diffuse, stain surrounding secretions, or spread beyond the intended site. Barium sulfate and other particles may differ in size, density, solubility, and adherence to mucus, all of which can influence the measured transport time. These limitations explain why visible non-charcoal markers are historically important but require careful standardization for clinical or experimental use.

Charcoal-based and activated charcoal methods

Charcoal-based methods belong to the broader category of visible marker techniques, but they have distinct physical and practical characteristics. Charcoal provides a dark, high-contrast marker that is insoluble, inexpensive, topically applicable, and visually detectable. These properties give charcoal-based testing a specific methodological identity within nasal mucociliary clearance assessment.

The contemporary interest in charcoal-based testing derives primarily from its characteristics as a visible marker rather than from its novelty. Historical studies demonstrate that charcoal can be used as a dark and readily detectable tracer, including in pediatric populations, while contemporary comparative work has renewed interest in examiner-observed charcoal transit [12-18]. These characteristics may reduce dependence on a taste-reported endpoint; however, they do not establish superiority over saccharin testing or other established methods.

Procedural standardization is likely to be important when charcoal-based testing is used clinically or experimentally. Relevant variables include marker composition and concentration, application amount, anatomical placement, patient position, observation interval, and endpoint definition. Nevertheless, the extent to which standardization improves reproducibility or diagnostic performance requires prospective evaluation.

Direct endoscopic visualization may provide a means of controlling marker placement and observing the endpoint, but its additional value has not yet been established in prospective comparative studies. Endoscopically controlled activated-charcoal testing should therefore be considered a methodological approach warranting further investigation, rather than an already validated enhancement of conventional charcoal testing.

Radiolabeled particle and scintigraphic techniques

Radiolabeled particle methods and scintigraphic techniques represent the more objective and technically demanding branch of nasal mucociliary clearance assessment. Their principle is based on the application of a radiolabeled tracer to the nasal mucosa and subsequent detection or imaging of its movement over time. These approaches reduce dependence on patient sensation and verbal reporting and can provide quantitative transport data.

Their main limitation is practical. Radiolabeled and scintigraphic methods require radioactive tracers, imaging or detection equipment, trained personnel, specialized protocols, and often cooperation between otorhinolaryngology and nuclear medicine. They are valuable in research and selected diagnostic contexts, but they are less suitable for routine office-based testing and less attractive for repeated assessment in children. Even when radiation exposure is low, the use of radiolabeled materials introduces additional practical and ethical considerations when applied to pediatric populations.

Modern ciliary function tests and primary ciliary dyskinesia diagnostics

Modern assessment of mucociliary function has expanded beyond the measurement of nasal transport time. Ciliary beat frequency analysis, high-speed video microscopy, nasal nitric oxide measurement, transmission electron microscopy, immunofluorescence, and genetic testing are now central to the diagnostic evaluation of primary ciliary dyskinesia and related disorders.

These techniques provide greater diagnostic depth but should not be treated as direct substitutes for nasal transport-time testing. A marker-based nasal clearance test measures the time required for material to move along the nasal mucosa toward a defined endpoint. In contrast, modern ciliary diagnostics evaluate the biological mechanisms that may underlie impaired clearance, including ciliary motion, ultrastructure, protein expression, nitric oxide biology, or genetic defects. Therefore, these approaches should be considered complementary rather than competing methods.

Comparative methodological analysis

The diversity of methods used to assess nasal mucociliary clearance reflects the absence of a universally ideal test. Saccharin testing is simple, inexpensive, and accessible but relies on a subjective, taste-reported endpoint. Dye-based and visible particle methods provide examiner-observable transport but remain sensitive to tracer properties, diffusion, and procedural standardization. Charcoal-based testing has historical and pediatric precedent as a visible-marker approach, while the additional value of combining activated charcoal with direct endoscopic control has not yet been established prospectively. Radiolabeled and scintigraphic techniques provide greater objectivity and quantification but require specialized resources. Modern ciliary diagnostics are essential in suspected primary ciliary dyskinesia but address different biological and diagnostic questions than those addressed by nasal transport-time testing.

Accordingly, these methods should be viewed as complementary tools selected according to the clinical or research objective rather than as directly interchangeable alternatives. Charcoal-based testing remains of methodological interest because it provides a visible, examiner-observed endpoint; however, standardized endoscopically controlled activated-charcoal testing should currently be regarded as a candidate methodology for prospective comparative evaluation, rather than as an established clinical advantage over existing methods.

Pediatric considerations in nasal mucociliary clearance testing

Pediatric nasal mucociliary clearance testing presents specific methodological challenges. In children, the choice of method is influenced not only by physiological relevance, but also by safety, tolerance, cooperation, anxiety, duration of the procedure, equipment requirements, reproducibility, and endpoint reliability. A method that is acceptable in cooperative adults may become less reliable in younger children if it requires sustained attention, taste recognition, precise verbal reporting, or repeated instruction during the examination.

This issue is particularly important because several pediatric upper airway conditions are associated with altered nasal mucociliary clearance. Adenoid hypertrophy has been investigated as a clinical model in which mechanical obstruction, chronic inflammation, mucus stasis, and impaired mucosal defense may interact. Maurizi et al. evaluated adenoid hypertrophy and nasal mucociliary clearance in children using the saccharin method and scanning electron microscopy, showing that pediatric adenoid disease was already being studied not only as an obstructive condition, but also as a functional mucociliary problem [25].

The same research group later evaluated 34 children with clinically and radiologically confirmed adenoid hypertrophy before and six months after adenoidectomy. Their study reported a significant increase in mucociliary clearance velocity and a decrease in binasal resistance after surgery, supporting the concept that adenoidectomy may improve not only airway patency but also nasal mucociliary function [26].

Subsequent pediatric studies further connected mucociliary clearance assessment with clinical symptoms and adenoid surgery. Arnaoutakis and Collins studied children undergoing adenoidectomy or adenotonsillectomy using saccharin and methylene blue testing before and after surgery and related mucociliary clearance to symptomatology. Their work placed mucociliary assessment in a clinically meaningful pediatric framework rather than treating it as an isolated physiological measurement [27]. Marusiakova et al. evaluated ciliary beat frequency in children with adenoid hypertrophy and reported impaired ciliary activity in clinically symptomatic children, supporting the broader interpretation that adenoid hypertrophy may be associated with altered mucociliary defense [28]. More recently, Berkiten et al. evaluated the effects of adenoid hypertrophy on mucociliary clearance and nasal cytology in children, reinforcing the clinical relevance of functional mucociliary assessment in pediatric nasal disease [29].

Against this pediatric background, the methodological limitations of subjective endpoint tests become more relevant. The saccharin transit time test may be simple and widely available, but it requires the child to understand the instructions, remain calm, avoid behaviors that could alter the test, recognize the taste stimulus, and report it accurately. Visible marker methods offer a potential advantage because the examiner can identify the marker or endpoint directly. The historical use of vegetal coal powder in children aged three to 12 years and charcoal dust testing in children before and after adenoidectomy demonstrate that charcoal-based visible marker methods have pediatric precedent [13,15].

The pediatric value of activated charcoal should be framed in this context. It does not eliminate all sources of variability, because children may still move, cry, cough, sniff, swallow repeatedly, or fail to tolerate nasal endoscopy. However, a visible dark marker may reduce one important source of variability: dependence on subjective taste perception and verbal reporting. These characteristics suggest that a visible charcoal marker may warrant further evaluation in selected pediatric otorhinolaryngology settings, particularly when subjective taste perception and verbal reporting may compromise test reliability.

The methodological niche of endoscopically controlled activated charcoal testing

The role of activated charcoal should be defined with precision. Historical studies establish that charcoal-based tracers have been used for nasal mucociliary transport assessment in adults and children, including before and after adenoidectomy, and that charcoal can serve as a dark, visible, insoluble marker [12-18]. These data support its historical feasibility and potential for examiner-observed endpoint detection, but not superiority over established methods.

The combination of a visible charcoal marker with procedural standardization and direct endoscopic visualization should therefore be regarded as a proposed methodological framework rather than an established clinical advantage. The reviewed literature does not contain prospective head-to-head evidence demonstrating that endoscopic control improves reproducibility, accuracy, or clinical utility when performing charcoal testing.

Future comparative studies should prospectively predefine marker preparation and concentration, application amount, anatomical placement, patient position, observation interval, endpoint definition, tolerability, reproducibility, and agreement with established methods. Endoscopy could then be evaluated specifically to determine whether it improves placement control and endpoint recognition rather than assuming such benefit a priori. Until such evidence is available, activated charcoal is best positioned as a candidate visible-marker approach for methodological research, particularly where dependence on subjective taste reporting is problematic, and not as a routinely recommended or universally superior clinical test.

Limitations of the review

This review has several limitations. It was designed as a narrative historical-methodological review rather than a systematic review; therefore, it does not claim to provide an exhaustive retrieval of all available publications. Although the literature search was structured and supplemented by reference-list screening, publication selection remained guided by relevance to the historical and methodological aims of the review, and selection bias cannot be completely excluded. A formal study-level risk-of-bias assessment was not performed. In addition, several historically important publications predate contemporary reporting standards and provide variable detail regarding participant selection, study protocols, and methodological quality.

The available literature is also heterogeneous with respect to study populations, tracer characteristics, application techniques, anatomical placement, endpoint definitions, and reported outcome measures, which limits direct comparison between methods and precludes meaningful quantitative synthesis. Evidence specifically evaluating standardized endoscopically controlled activated charcoal testing remains limited; consequently, its potential methodological advantages should be regarded as hypotheses requiring prospective comparative validation, rather than as established clinical benefits.

## Conclusions

Nasal mucociliary clearance assessment has evolved from early direct observation and simple marker-based techniques to saccharin testing, visible particle and charcoal-based methods, radiolabeled and scintigraphic approaches, and modern specialized ciliary diagnostics. No single method is universally optimal; each differs in objectivity, technical complexity, cost, patient cooperation requirements, and the physiological or clinical question it addresses. Traditional nasal transport tests and specialized investigations for ciliary disorders should therefore be regarded as complementary rather than interchangeable approaches. Charcoal-based testing has a documented historical and pediatric precedent as a visible-marker method for nasal mucociliary transport assessment. Its practical characteristics, including visual detectability, topical application, and reduced dependence on a taste-reported endpoint, justify continued methodological interest.

However, the additional value of combining activated charcoal with standardized endoscopic control has not yet been established in prospective comparative studies. Accordingly, this approach should currently be considered a candidate methodological framework rather than a routinely recommended or superior clinical test. Future research should prospectively compare standardized activated-charcoal protocols with established mucociliary clearance methods and assess reproducibility, agreement, accuracy, feasibility, patient tolerance, and age-related applicability. Such studies are particularly relevant in pediatric otorhinolaryngology, where cooperation and reliable subjective endpoint reporting may influence test performance. Prospective validation will be necessary to determine whether endoscopically controlled activated-charcoal testing has a defined role in clinical practice or research.
